# 

**Published:** 1875-01-01

**Authors:** 


					﻿No. I.
JANUARY 1, 1875
Vol. XVI.
T'ZEEZE
MEDICAL EXAMIKEB:
mmi-||onthlg <|fourLnal of jWral
EDITED BY
N. S. DAVIS, M. D., and F. H. DAVIS, M. D.
Subscription Price, Three Dollars per Annum, in advance
Single Numbers, Fifteen Cents.
CHICAGO
PUBLISHED AT 65 El. RANDOLPH STREET.
				

